# General Charities

**Published:** 1918-12-21

**Authors:** 


### General Charities.

**DR. BARNARDO'S HOMES, STEPNEY CAUSEWAY, E. 1.**—Child life is the nation's greatest asset. 6,782 children admitted since war broke out, a large proportion being the children of our brave sailors and soldiers. General support for food, which gets dearer each day, for the large family of over 7,000 children is appealed for. Especially will gifts of all kinds—money, clothing, blankets—be welcomed for Christmastide. Many old Barnardo boys and girls have done splendid war service. An interesting little book, describing the work of the Homes, can be obtained on application to the Secretary.

**EAST-END MOTHERS' LYING-IN HOME, COMMERCIAL ROAD EAST.**—During 1918 six hundred and fifty babies were born in the Home. The year's work in the Home stands out successfully, although anxiety for absent husbands, air raids, and the long food queues are not the ideal circumstances to be chosen for motherhood. Yet these things existed, and it was heartbreaking to send mothers and infants out from the care and comfort of the Home to their own impossible shelters.

**THE MENTAL AFTER-CARE ASSOCIATION, CHURCH HOUSE, DEAN'S YARD, WESTMINSTER, S.W. 1.**—This Association has during the past year assisted a very large number of shell-shock and air-raid patients, which has very much increased the work. The results have been most gratifying, and in nearly all cases those who have passed through the hands of this Association have again become self-supporting, and, in almost all instances, are doing well. Miss Vickers is the Secretary.

**METROPOLITAN CONVALESCENT INSTITUTION, 14 VICTORIA STREET, S.W.**—Although the support to this institution has been well maintained during the past year, it has not in any sense been commensurate with the large increase in the cost of upkeep. The institution is in great demand for war pensioners and already a large number of ex-soldiers whose health has been impaired by wounds or privations have received great benefit by a stay at the Walton or Bexhill branches.

**NATIONAL TRAINING SCHOOL FOR DISTRICT MIDWIVES (BRITISH HOSPITAL FOR MOTHERS AND BABIES, WOOLWICH).**—This is one of the most urgent necessities of reconstruction—a training school where such pupil midwives as are destined to work among the labouring classes may receive, at small cost to themselves, a year's thorough training in all that concerns the welfare of mothers and infants. The British Hospital is now working in cramped quarters and under great disadvantages. The hospital has, with some difficulty, maintained itself free of debt. Miss Alice S. Gregory, Hon. Secretary.

**ROYAL HOSPITAL AND HOME FOR INCURABLES, PUTNEY HEATH.**—£40,000 is needed annually to provide a home for incurables above the pauper class, and for those having a home but no means of support, a pension of £20 a year. Mr. Charles Cutting, Secretary. The provision of a nurses' home for about 80 or 90 residents is under consideration. Offices, Bond Court House, Walbrook, London, E.C. 4.

